# Antibacterial Properties of Citric Acid/β-Alanine Carbon Dots against Gram-Negative Bacteria

**DOI:** 10.3390/nano11082012

**Published:** 2021-08-06

**Authors:** Anju Pandey, Asmita Devkota, Zeinab Yadegari, Korsi Dumenyo, Ali Taheri

**Affiliations:** 1 Department of Agricultural and Environmental Sciences, College of Agriculture, Tennessee State University, 3500 John A Merritt Blvd, Nashville, TN 37209, USA; pandeyanju931@gmail.com (A.P.); devkota.asmita1996@gmail.com (A.D.); cdumenyo@tnstate.edu (K.D.); 2 Department of Life and Physical Sciences, Fisk University, 1000 17th Ave N, Nashville, TN 37208, USA; zyadegar@gmail.com

**Keywords:** antimicrobial, multiple drug resistance, MDR, carbon dots

## Abstract

While multi-drug resistance in bacteria is an emerging concern in public health, using carbon dots (CDs) as a new source of antimicrobial activity is gaining popularity due to their antimicrobial and non-toxic properties. Here we prepared carbon dots from citric acid and β-alanine and demonstrated their ability to inhibit the growth of diverse groups of Gram-negative bacteria, including *E. coli*, *Salmonella*, *Pseudomonas*, *Agrobacterium*, and *Pectobacterium* species. Carbon dots were prepared using a one-pot, three-minute synthesis process in a commercial microwave oven (700 W). The antibacterial activity of these CDs was studied using the well-diffusion method, and their minimal inhibitory concentration was determined by exposing bacterial cells for 20 h to different concentrations of CDs ranging from 0.5 to 10 mg/mL. Our finding indicates that these CDs can be an effective alternative to commercially available antibiotics. We also demonstrated the minimum incubation time required for complete inhibition of bacterial growth, which varied depending on bacterial species. With 15-min incubation time, *A. tumefaciens* and *P. aeruginosa* were the most sensitive strains, whereas *E. coli* and *S. enterica* were the most resistant bacterial strains requiring over 20 h incubation with CDs.

## 1. Introduction

Due to their cell wall composition, Gram-negative bacteria are reported to be more resistant to antibiotics compared to Gram-positive bacteria [1,2]. Gram-negative bacteria possess an extra protective outer membrane of lipopolysaccharides that limits the entry of certain antibiotics. The majority of commercially available antibiotics follow two pathways to enter the Gram-negative bacterial cell. Hydrophobic antibiotics use lipid-mediated pathways, whereas hydrophilic antibiotics enter the cells using diffusion porins [3]. However, due to frequent modifications in lipid and protein compositions and porins in the outer membrane of Gram-negative bacteria, they are selective against larger hydrophilic antibiotics and therefore are more resistant to antibiotics. In addition, due to intensive prescription of antibiotics, many bacteria are becoming multi-drug resistant (MDR) [4]. Hence, there is a need for alternative antibiotics with different chemistries for the treatment of infections caused by Gram-negative bacteria. Recently, nanomaterials have gained popularity as novel antimicrobial agents and also for nano-sensing and nano-delivery of biomolecules into the cells especially because of their prominent characteristics, such as nanostructure (1–100 nm), biocompatibility, and auto-fluorescence (easy to track inside cells) [5]. Apart from these characteristics, carbon dots are water-soluble and less toxic to mammalian cells than traditional antibiotics. Moreover, they have higher chemical stability and simpler preparation steps [6,7,8,9,10]. Because of these exceptional characteristics, carbon dots are widely used in the fields of biotechnology, energy, catalysts, biological labeling, bioimaging, gene transfer, and drug delivery at the target site [8,9,11]. Among different nanoparticles, carbon dots (CDs) are popular as antimicrobial agents due to their abundance and minimal toxicity [5,12]. They are effective against different microbes, such as bacteria, fungi, and viruses [13]. CDs can inhibit bacterial growth upon direct contact with bacterial cells because of the release of reactive oxygen species (ROS), which, in turn, leads to impairment of biomolecules and cell death [13,14]. However, the exact details of the inhibitory mechanism of different carbon dots need to be further investigated.

Here, we demonstrate the synthesis of CDs in a commercial microwave (700 W) using citric acid as a carbon source and β-alanine as a surface passivator. These CDs have previously been used for drug delivery and optical monitoring [8]. In this report, we demonstrate the potential application of β-alanine based CDs as novel antimicrobial agents against a diverse group of Gram-negative bacteria, including *E. coli*, *Pseudomonas*, *Salmonella*, *Agrobacterium*, and *Pectobacterium*.

## 2. Materials and Methods

All the reagents for CD synthesis, including monohydrate citric acid (Cat#A104-500) and β-alanine (Cat#AAA166650I), were purchased from Thermo Fisher Scientific (Waltham, MA, USA). Bacteriological agar and yeast extract from IBI Scientific (Dubuque, IA, USA) and Tryptone and sodium chloride for the preparation of Luria broth and agar media for culturing *E. coli*, *Pectobacterium,* and *Salmonella* were purchased from MIDSCI^TM^ (Valley Park, MO, USA). Protease peptone, dibasic potassium phosphate, magnesium sulfate heptahydrate, glycerol, and bacteriological agar were used to prepare King’s media B (KB) for culturing *Pseudomonas*. Yeast extract, Bacto-peptone, and sodium chloride were also used to prepare YEP media for *Agrobacterium* culture.

### 2.1. Synthesis of Carbon Dots (CDs) 

**Figure 1:**
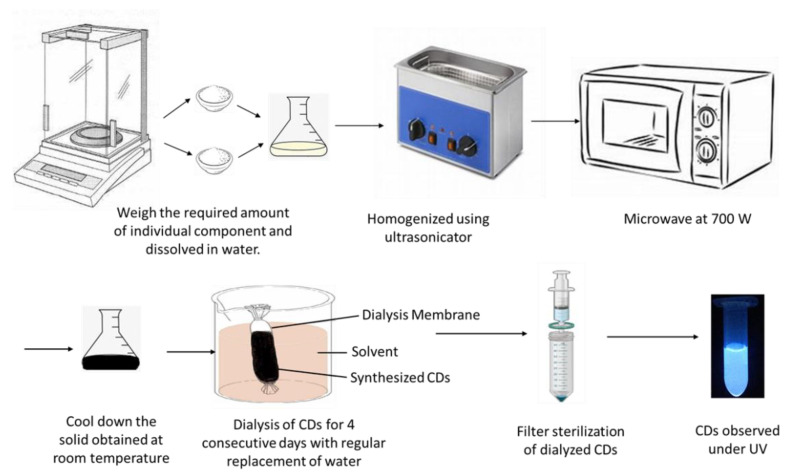
Diagram displaying the synthesis and purification of carbon dots using a microwave oven.

Carbon dots were synthesized using citric acid and β-alanine according to a previous report [8] with a slight modification, outlined in Figure 1. We used citric acid monohydrate (Fisher #A104-500, Pittsburg, PA, USA) as a carbon source instead of anhydrous citric acid (Fisher #BP339-500, Pittsburg, PA, USA) at the same molar ratio and observed similar results. Briefly, CDs were synthesized by mixing 1:2 molar ratio of citric acid and β-Alanine, where one gram of citric acid was mixed with 0.9 g of β-Alanine in 10 mL of distilled water (pH 3) in a conical flask. The mixture was homogenized using an Ultrasonicator (Ultrasonic Cleaner FS30, Fisher Scientific, Pittsburg, PA, USA) until completely dissolved and then heated for 3 min in a commercial microwave oven (Model#JES2251SJ02, GE Appliance, Louisville, KY, USA) at 70% power level to proceed to carbonization and surface passivation. The obtained brownish solid was then dissolved in 10 mL of distilled water (pH 3).

### 2.2. Purification of CDs

Purification of CDs was performed by dialysis using Spectra/Por^®^ 7 Dialysis Membrane with 11.5 mm diameter (#08-700-198, Fisher Scientific, Pittsburg, PA, USA). The water tank was refreshed every 2 h for the first initial 8 h, then once a day for another 4 days. Purified CD solutions were further passed through a 0.22 µm pore size filter.

### 2.3. Preparation of Bacteria Strains

*Escherichia coli* DH5α, *Pectobacterium carotovorum* Ecc7, *Agrobacterium tumefaciens* EHA101, *A. rhizogenes* K599, *Pseudomonas syringae* pv. *tomato* DC3000, and *Salmonella enterica* subsp *enterica* serovar *typhimurium* 13,311 were used in this study. *A. tumefaciens*, *A. rhizogenes*, *P. syringae* pv. *tomato*, *P. carotovorum* were cultured overnight in YEP, King’s B, and Luria Broth, respectively, at 28 °C. Similarly, *E. coli* and *S. enterica* subsp *enterica* were grown on Luria Broth at 37 °C. OD_600_ was adjusted to 0.5 by subculturing the overnight culture and harvesting at the logarithmic growth phase. Five milliliters of bacterial culture was centrifuged and washed three times with sterile distilled water, resuspended in 1 mL of 15% glycerol, aliquoted in 50 µL in 0.65 mL tubes, and stored at −80 °C for long-term use.

### 2.4. Characterization of CDs

Fourier-transform infrared (FTIR) spectroscopy analysis was performed to confirm the functional groups of CDs. ATR-FTIR analysis was carried out using a Perkin Elmer Frontier Infrared spectrometer equipped with a liquid nitrogen-cooled MCT-A (mercury cadmium telluride) detector and an optics compartment purged with CO_2_- and H_2_O-free air delivered by a Balston-Parker air purger. Freeze-dried CDs were used for FTIR analysis, and FTIR spectra were recorded between 600 and 3800 cm^−1^. Photoluminescence properties, such as absorbance, excitation, and emission wavelength, of the CDs were recorded using a Synergy H1 Hybrid Multi-Mode Microplate Reader (BioTek, Winooski, VT, USA). The Zetasizer nano ZS (Malvern Panalytical Inc., Westborough, MA, USA) was used to measure the electrostatic charges carried by CDs.

### 2.5. Antimicrobial Study

The antimicrobial activities of CDs were evaluated by incubating bacterial cells with different concentrations of CDs and different time intervals. The Agar plate well-diffusion method was used to examine the antimicrobial activity of CDs diluted at different concentrations [15]. Agar plates were inoculated with 50 µL of bacterial suspension harvested at OD_600_ = 0.5 (approximately 4 × 10^8 CFU) using sterile glass beads. Then, with the help of a cork borer, a well of 0.6 mm diameter was prepared on the inoculated plates. Forty microliters of CDs solution was added to each well and incubated overnight at 28 or 37 °C depending on the bacterial strain. To determine the minimum incubation time required for the complete inhibition of bacterial cells, 50 µL of cells (harvested at OD_600_ = 0.5) were mixed with 5 µL of freshly prepared CDs (~19 mg/mL concentration) and incubated for different time intervals ranging from 1 h to 16 h. The incubation time with no bacterial growth was recorded as complete inhibition of cell growth.

### 2.6. Effect of Light on Antimicrobial Properties of CDs

To evaluate the effect of light on the antimicrobial properties of CDs, a mixture of 50 µL of bacterial suspension harvested at OD_600_ = 0.5 and 5 µL of freshly prepared CDs (19 mg/mL) were incubated for different time intervals under different light conditions (light and dark condition).

### 2.7. Statistical Analysis

The diameter of the inhibition zone for each bacterium was tabulated and analyzed using R software (V3.6.3). The mean of each treatment was calculated from three biological replicates, and a post hoc test was conducted by the least significant difference (LSD) *t*-test.

## 3. Results and Discussion

### 3.1. Characterization of CDs

**Figure 2:**
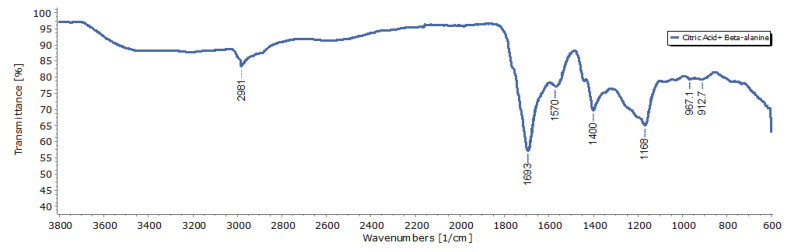
FTIR spectrum of Citric acid/β-alanine.

Fourier-transform infrared (FTIR) spectroscopy was conducted to characterize the chemical functional groups on the CDs (Figure 2). The FTIR results showed peaks at 1168 cm^−1^ (C-O) of the carbonyls group, 1400 cm^−1^ (C-N) of the Nitrile group, 1693 cm^−1^ (C=O) of ketone group, and 2981 cm^−1^ (C-H) of Alkane group. Successful passivation of β-alanine was indicated by the presence of the 1400 cm^−1^ peak (C-N stretching) [8]. Obtained peaks were in accordance with a previous report, suggesting the successful synthesis of CDs [8].

**Figure 3:**
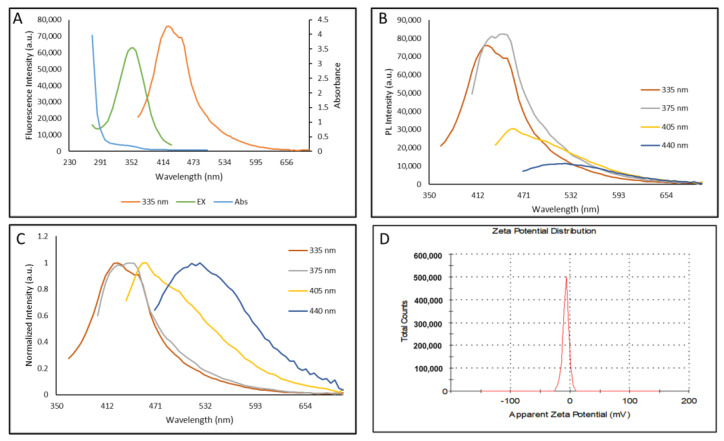
Photoluminescence absorbance, emission, and excitation spectra of carbon dots (CDs). (**A**) Absorbance, emission, and excitation spectra of CA+β-alanine recorded from 230–700 nm in 5 nm increments. (**B**) Emission spectrum showing excitation dependent emissions at different excitation wavelengths ranging from 335 nm to 440 nm; (**C**) Normalized emission spectrum of CDs showing variation in the intensity of spectrum at different excitation wavelengths, (**D**) zeta-potential of CA+β-alanine at pH 3.

Photoluminescence properties of CDs synthesized in this study showed two characteristic absorbance peaks at 275 nm and 350 nm. These peaks are respectively due to the sp^2^-carbon network and n–π* transition of surface carbonyl groups [16] (Figure 3A). Their fluorescence emission profile showed excitation-dependent emission spectra of these carbon dots. The excitation wavelength increment from 335 to 440 nm resulted in a shift in the emission peak along with a reduction in the peak intensity (Figure 3B) [17]. The maximum emission peak for these CDs was 445 nm with excitation at 375 nm, which is similar to other carbon dots [18] (Figure 3C).

CDs synthesized in this study showed a zeta-potential value of −8.09 ± 5.68 mV measured by the Malvern Zetasizer Nano-ZS ZEN 3600 (Figure 3D). This negative charge provides sufficient colloidal stability to CDs and is due to the presence of two negatively charged functional groups (C=O and C-O) at the surface of the synthesized CDs [19,20].

### 3.2. Interaction between CDs and Bacterial Cells

**Figure 4:**
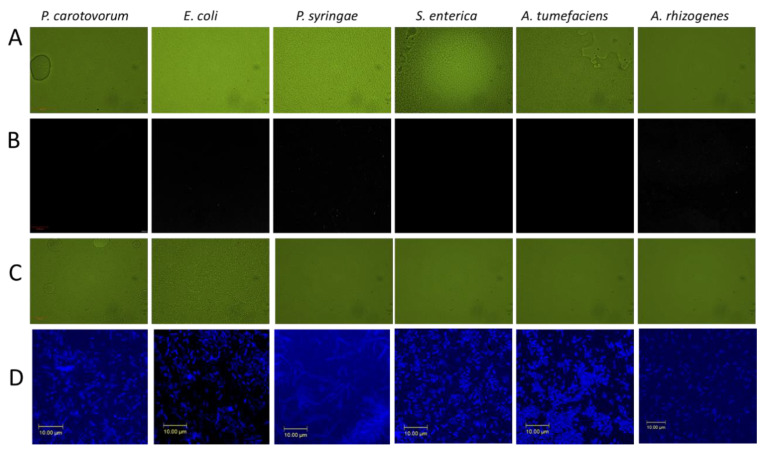
Laser confocal scanning microscopy of CDs interaction with bacteria. (**A**) Cells only in bright field. (**B**) Cells only in confocal microscope. (**C**) Cells incubated with CA+β-alanine CDs for 1 h in bright field. (**D**) Cells incubated with CA+β-alanine CDs for 1 h under confocal microscope; magnification = 63× (Scale bar indicates 10 µm).

The interaction between cells and CDs was confirmed by confocal microscopy. For this purpose, 5 µL of CA+β-alanine CDs (19 mg/mL) and 50 µL of bacterial cells (O.D_600_ = 0.5) were incubated for 1 h. Cells were centrifuged and washed three times with sterile water to remove free or unbound extracellular carbon dots. Interaction and bonding of CDs with bacterial cell surface was confirmed under laser confocal scanning microscopy (LCSM) (Figure 4).

### 3.3. Antibacterial Activity

**Figure 5:**
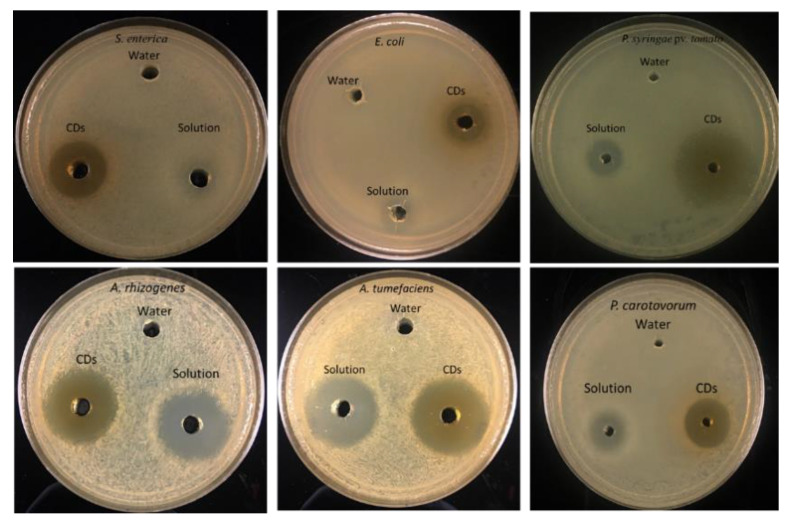
Growth inhibition of bacteria by citric acid/β-alanine CDs. The indicated Gram-negative bacteria were seeded on the agar media, and holes were made with cork-borer (0.6 mm in diameter). The bottom of the holes was sealed with 0.5% agar, and 40 µL of CDs (19 mg/mL) was added to each well.

**Figure 6:**
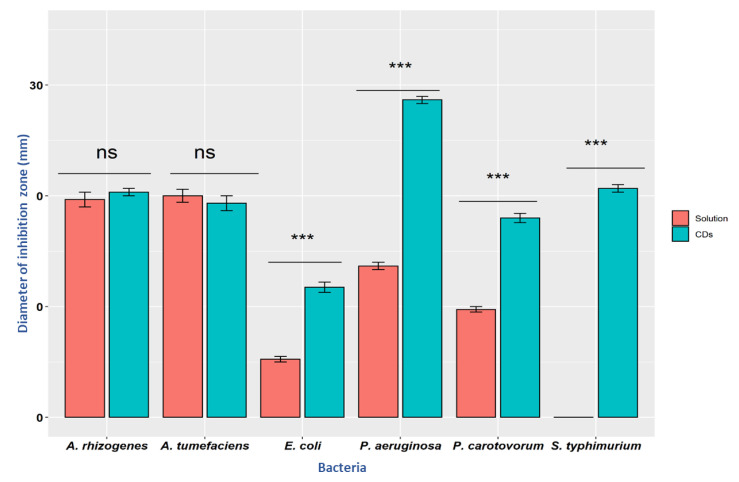
Growth inhibition of different Gram-negative bacteria by the CDs (19 mg/mL) and their pre-cursor solution (Citric acid and beta-alanine). Values are mean (from three replicates) ± standard deviation. Two way ANOVA, *** *p* = 0.001: ns = non-significant.

The antimicrobial effect of synthesized CDs was studied on five Gram-negative bacteria using the agar-plate diffusion method. Both solutions of citric acid + β-alanine (precursors used to prepare the CDs) and synthesized CDs were evaluated for their antimicrobial activities. Bacterial cells were plated on each plate, and diffusion wells were created on each plate using a cork borer. Each well in the agar plate was then filled with 40 µL of 19 mg/mL of CDs and plates were transferred into an incubator with appropriate temperature for each bacteria. The results showed that the antimicrobial capacity was enhanced in CDs compared to the solution (Figure 5). CDs were most effective against *E. coli, Salmonella, Pectobacterium,* and *Pseudomonas* (Figure 6). However, both Citric acid + Β-alanine solution and CDs were found to be equally effective against both strains of *Agrobacterium* (Table 1).

**Figure 7:**
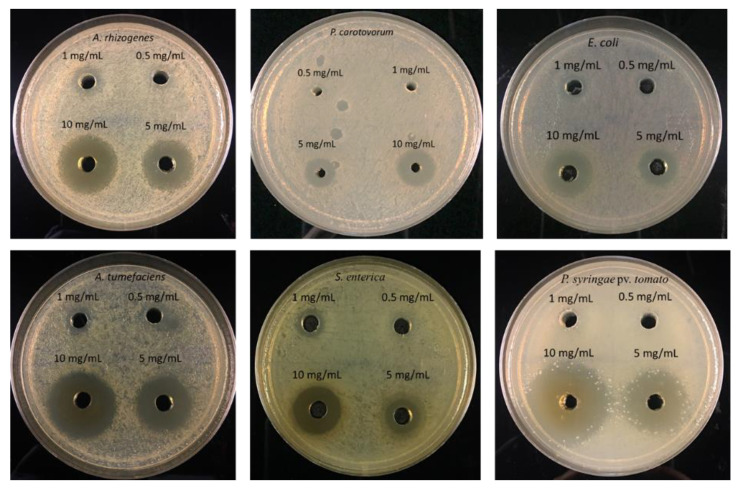
MIC of CDs as determined by agar-plate well diffusion method.

**Figure 8:**
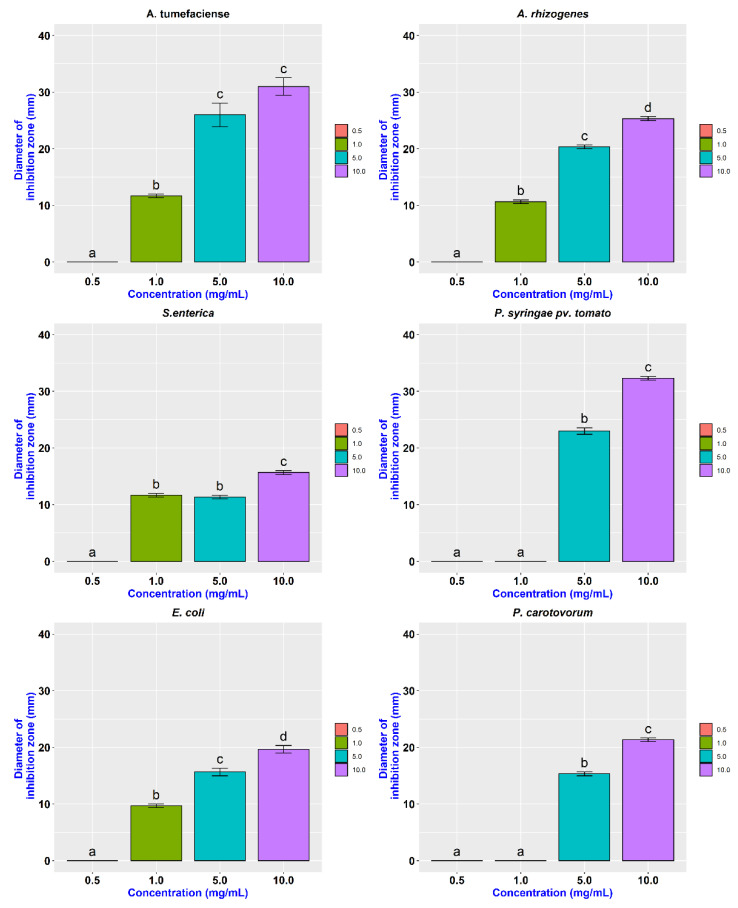
MIC of CDs against different Gram-negative bacteria. Values are mean (from three replicates) ± standard deviation. Means of diameter of inhibition zones falls into different groups a, b, c and d indicating significant variation among different concentration of CDs.

Similarly, the agar-plate well diffusion method was used to determine the Minimum Inhibition Concentration (MIC) of CDs. Among different concentration of CDs (0.5 mg/mL, 1 mg/mL, 5 mg/mL, and 10 mg/mL) used in this study, 1 mg/mL was found to inhibit the growth of *Agrobacterium, Salmonella,* and *E. coli* (Figure 7). Whereas a concentration of 5 mg/mL resulted in distinct inhibition zones for *Pectobacterium* and *Pseudomonas,* and they displayed more resistance to lower concentrations (Table 2). Carbon dots were most effective against *Pseudomonas* followed by *A. tumefaciens* with a mean inhibition zone of 32.33 mm and 31 mm at 10 mg/mL concentration, respectively (Figure 8).

### 3.4. Minimum Incubation Time for Complete Inhibition of Bacterial Growth

**Figure 9:**
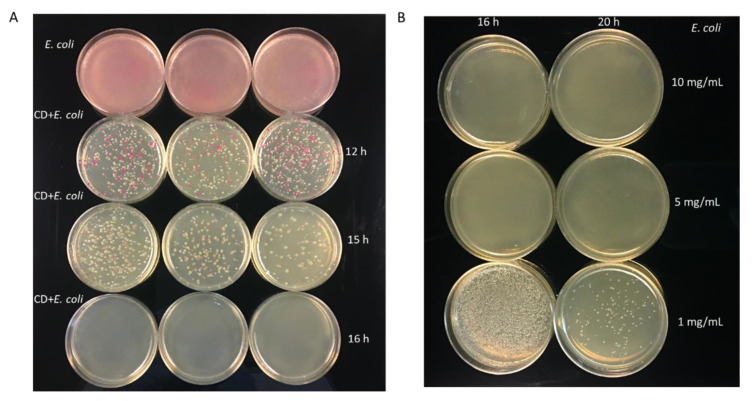
Antimicrobial activity of CDs against *E. coli* at different concentrations and incubation intervals. (**A**). Growth inhibition of *E. coli* in the presence of CDs (19 mg/mL) at different incubation time. (**B**). Growth inhibition of *E. coli* at different concentrations of CDs and incubation times.

**Figure 10:**
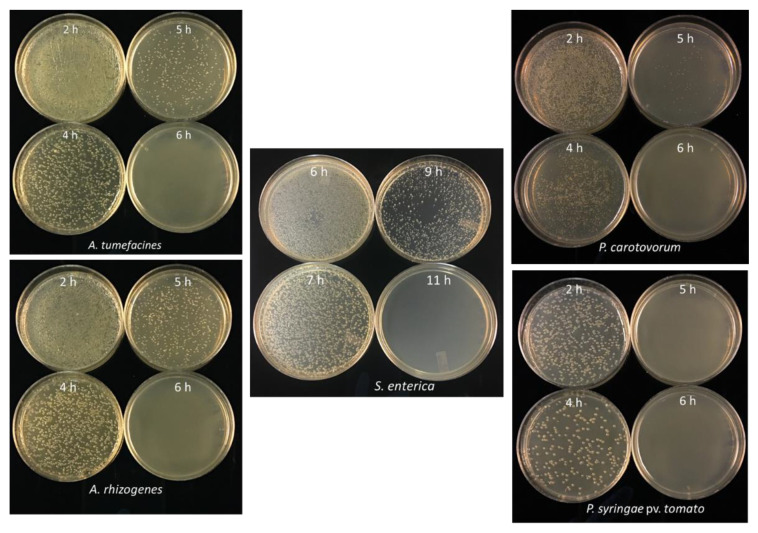
Complete growth inhibition in different bacterial species with b-Alanine and citric acid CDs (19 mg/mL).

To determine the minimum incubation time required for complete inhibition of bacterial growth, 50 µL of bacterial cell suspension was incubated with 5 µL (19.0 mg/mL) of freshly synthesized CDs and incubated for different time intervals. Different incubation times are required for complete growth inhibition of different bacterial species. For *E. coli,* a minimum incubation time of 16 h was required to completely inhibit the bacterial cell growth (Figure 9A). Similarly, the MIC of CDs against *E. coli* was determined along with different concentrations of CDs in which the complete inhibition was only observed at 5 mg/mL and 10 mg/mL when incubated for 16 h (Figure 9B). In most other bacteria, complete inhibition was obtained after 5–6 h of incubation with CDs (19 mg/mL), except *S. enterica* that required 11 h of incubation (Figure 10).

We tested the antimicrobial activity of synthesized CDs and identified its inhibitory effects against several Gram-negative bacteria. Unlike Gram-positive bacteria, Gram-negative bacteria possess an extra protective layer of lipopolysaccharides which renders them more resistant to commercially available antibiotics. However, the negatively charged CDs might bind to divalent cations Ca^2+^ and Mg^2+^ that are present at the lipid layer of bacterial cells [21,22]. This interaction could lead to the breakage of phosphate group bonds at the membrane lipid, which leads to destabilization of lipopolysaccharide, increased membrane permeability, leakage of cytoplasmic fluid, and cell death [23].

### 3.5. Effect of Light on Antimicrobial Activity

**Figure 11:**
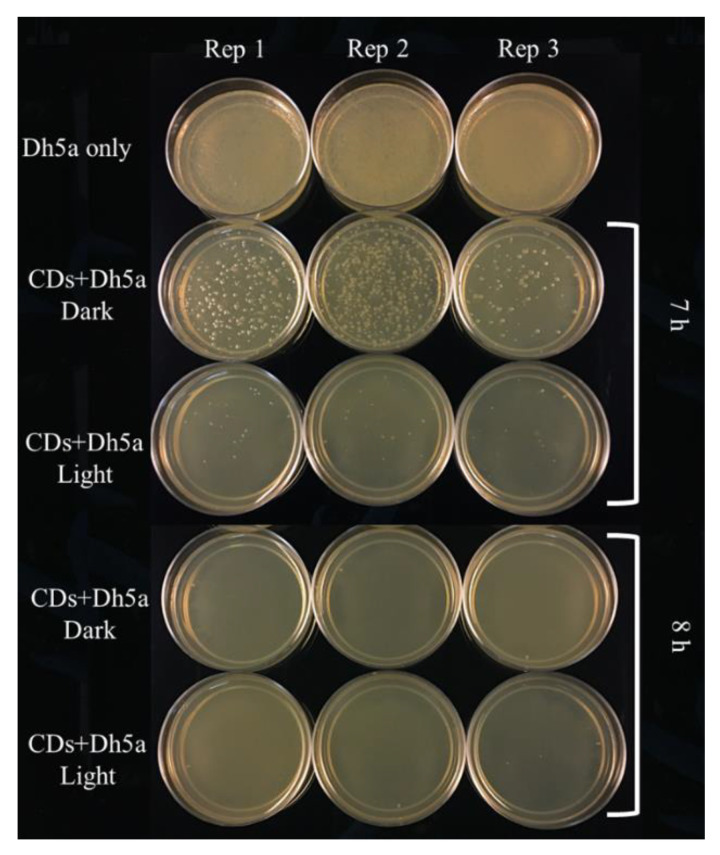
Effect of light on antimicrobial properties of CA+β-alanine carbon dots. Dh5 cells were incubated with 5 µL of CDs (19 mg/mL) for 7 and 8 h.

The effect of light on antimicrobial properties of CDs was investigated by incubating a mixture of bacterial cells and CDs in light or dark conditions at different time intervals (Figure 11). The results were in accordance with Dong et al., 2020, indicating that antimicrobial properties of CDs are light-dependent [24,25,26,27]. Photoactivation of CDs under ambient room lighting leads to the production of ROS in bacterial cells upon their contact and causes growth inhibition and cell death. Even though light affects the antimicrobial properties of CDs synthesized from citric acid and beta-alanine, our results indicate that longer incubations in the dark are also able to prevent bacterial growth indicating that these CDs also carry antimicrobial properties that are not light-dependent. A recent report on cytotoxic side effects of photodegraded carbon dots on normal and cancerous human cells [28] indicates a demand for CDs that do not require photoactivation to gain antimicrobial properties. Hence, CDs synthesized from citric acid +beta-alanine can be an alternative for antimicrobial photo-independent carbon dots.

## 4. Conclusions

In this report, we evaluated the antimicrobial effects of CA+β-alanine carbon dots against a diverse group of Gram-negative bacteria and demonstrated that these carbon dots can be considered as a novel strategy for the fight against Gram-negative bacteria. The emerging issue of MDR can be addressed using nanomaterials with an antimicrobial capacity. However, further studies are required to understand the mechanisms involved in the antibiotic effect of these CDs, their interaction with the cell surface, their toxicity against human cells, and their efficacy against Gram-positive bacteria. It is also important to understand their interaction with bacterial cell surface using other techniques, such as X-ray photoelectron spectrometer (XPS) [29], in understanding the antimicrobial mechanisms and confirming whether this behavior is solely due to the electrostatic interactions between the protonated forms CDs and the lipids of the bacterial cell membrane or their ability to generate reactive oxygen species (ROS).

## Figures and Tables

**Table 1:** Growth inhibitory effects of CDs on Gram-negative bacteria as determined by the agar-plate diffusion method.

Bacteria	Inhibition Zone Diameter (mm)		
	Water	Solution	Carbon Dots
*P. carotovorum*	0.00 ± 0.00	9.75 ± 0.50	18.00 ± 0.82
*E. coli*	0.00 ± 0.00	5.25 ± 0.50	11.75 ± 0.96
*P. syringae* pv. *Tomato*	0.00 ± 0.00	13.67 ± 0.58	28.67 ± 0.58
*A. tumefaciens*	0.00 ± 0.00	20.00 ± 1.00	19.33 ± 1.15
*A. rhizogenes*	0.00 ± 0.00	19.67 ± 1.15	20.33 ± 0.58
*S. enterica* subsp *enterica* serovar *Typhimurium*	0.00 ± 0.00	0.00 ± 0.00	20.67 ± s0.58

Values are mean (from three replicates) ± standard deviation.

**Table 2:** MIC of CDs against different Gram-negative bacteria.

Bacteria	Concentration of CDs			
	0.5 mg/mL	1 mg/mL	5 mg/mL	10 mg/mL
*A. tumefaciens*	0.00 ± 0.00	11.67 ± 0.47	26.00 ± 2.94	31.00 ± 2.16
*A. rhizogenes*	0.00 ± 0.00	10.67 ± 0.47	20.33 ± 0.47	25.33 ± 0.47
*S. enterica*	0.00 ± 0.00	11.67 ± 0.47	11.33 ± 0.47	15.67 ± 0.47
*P. syringae pv tomato*	0.00 ± 0.00	0.00 ± 0.00	23.00 ± 0.82	32.33 ± 0.47
*E. coli*	0.00 ± 0.00	9.67 ± 0.47	15.67 ± 0.94	19.67 ± 0.94
*P. carotovorum*	0.00 ± 0.00	0.00 ± 0.00	15.33 ± 0.47	21.33 ± 0.47

Values are mean (from three replicates) ± standard deviation.

## Data Availability

All the data relevant to this study are provided in tables and figures. Further information can be requested from the corresponding author.
